# Liquid Chromatography Coupled with Linear Ion Trap Hybrid OrbitrapMass Spectrometry for Determination of Alkaloids in *Sinomeniumacutum*

**DOI:** 10.3390/molecules23071634

**Published:** 2018-07-04

**Authors:** Jinjun Shan, Xia Zhao, Cunsi Shen, Jianjian Ji, Jianya Xu, Shouchuan Wang, Tong Xie, Wenjun Tong

**Affiliations:** 1 Jiangsu Key Laboratory of Pediatric Respiratory Disease, Institute of Pediatrics, Nanjing University of Chinese Medicine, Nanjing 210023, China; jshan@njucm.edu.cn (J.S.); zhaoxiahy@126.com (X.Z.); cunsishen@126.com (C.S.); njustream@163.com (J.J.); jianyaxu@126.com (J.X.); wscnj@126.com (S.W.); 2 Jiangsu Collaborative Innovation Center of Biomedical Functional Materials, Jiangsu Key Laboratory of Biomedical Materials, School of Chemistry and Materials Science, Nanjing Normal University, Nanjing 210023, China

**Keywords:** alkaloids, fragmentation pattern, morphinan, *Sinomeniumacutum*

## Abstract

The characterization of alkaloids is challenging because of the diversity of structures and the complicated fragmentation of collision induced structural dissociation in mass spectrometry. In this study, we analyzed the alkaloids in *Sinomenium acutum* (*Thunb.*) *Rehderet Wil* by high resolution mass spectrometry. Chromatographic separation was achieved on a Phenomenex Kinetex C18 (2.1 mm × 100 mm, 2.6 μm) column with a mobile phase consisting of acetonitrile and water (0.1% formic acid) under gradient elution. A total of 52 alkaloids were well separated and 45 of them were structurally characterized, including morphinans, aporphines, benzylisoquinolines, and protoberberines. Specially, mass spectrometric study of the morphinan alkaloids were explicitly investigated. Electrostatic potential plot from simulation was calculated for determination of protonation sites. Further fragmentation analysis suggested that the C_3_H_7_N, CH_4_O, and H_2_O elimination was displayed in MS^2^ spectrum. These fragmentation pathways are universal for morphinan alkaloids having methoxy substituted cyclohexenone or cyclohexadienone moieties. Additionally, for nitrogen oxides, an ion-neutral complex intermediate is involved in the fragmentation process, generating additional oxygenated ions. All these results provided the universal rules of fragmentation used for detection of alkaloids, and will be expected to be highly useful for comprehensive study of multi-components in the herbal medicine analysis.

## 1. Introduction

The field of herbal medicines involves a chemical library containing thousands of chemical compounds. A large group of these compounds contain nitrogen, with the majority falling within the group of alkaloids. These naturally occurring nitrogen-containing compounds often possess strong potency, even strong toxicity [1,2]. Examples of these compounds include ephedrine, berberine, paclitaxel, morphine, and scopolamine. The structural characterization of these alkaloids is of particular importance. 

High resolution hybrid mass spectrometry (MS) is an excellent tool for the structural characterization of natural compounds. Flavones, saponins, anthraquinones, and phenylpropanoids have all been successfully analyzed by high-resolution hybrid MS [3]. Many practical methods have been proposed to assist structural characterization, including the fragment ion scan, neutral loss scan, and mass defect strategy; most of these studies have focused on fragmentation and/or accurate mass measurement to characterize unknown structures. The characterization of herbal medicines has been investigated extensively by MS techniques in recent years [4,5,6,7]. Nevertheless, the structural diversity of alkaloids still poses a great challenge to their characterization, as numerous inherent structural isomers exist for many compounds. In addition, some alkaloids cannot be identified by methods tracing fixed MS products in collision induced dissociation (CID), although these have demonstrated efficacy for compounds such as saponins and flavones. Tiny changes in the structure may cause differences in the MS^n^ spectra; for example, the MS^2^ spectra of quaternary protoberberine alkaloids with methoxy groups at C9 and C10 differ from those of alkaloids with methoxy groups at C10 and C11. Thus, further efforts are necessary to demonstrate the fragmentation behaviors of these alkaloids.

*Sinomenium acutum* (*Thunb*.) *Rehderet Wils* (*S. acutum*) has long been employed as a clinical treatment for arthritis [8]. Alkaloids are considered the main active components for this purpose [9]. Although the effectiveness of alkaloids has been clinically confirmed, explicit component profiling remains lacking, especially for major ingredients such as sinomenine, isosinomenine, and other related morphinan alkaloids. Zhang [10] and Raith [11] reported on the MS fragmentation of heroin-related alkaloids, indicating a fundamentally different MS fragmentation behavior. Common fragmentation patterns such as the retro-Diels-Alder and McLafferty rearrangements cannot explain the products generated in CID. Therefore, owing to the distinctive structures of morphinan alkaloids and the complicated MS^n^ spectra, fragmentation behavior has become an independent topic of importance in MS research. In this study, we analyzed the fragmentation patterns of the morphinan alkaloids and effectively analyzed the alkaloids in *S. acutum* with the aim of determining new rules or universal methods to analyze these complex alkaloids.

## 2. Results and Discussion

**Figure 1:**
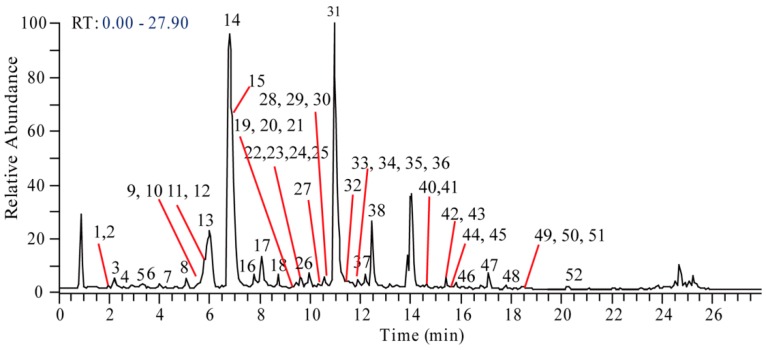
The total ion chromatogram (TIC) of the *S.acutum*extract.

To improve the overall performance of the chromatographic separation and sensitivity of MS detection, 0.1% formic acid was added in the mobile phase. A gradient elution program was further established and optimized to ensure better separation and fast elution of all the analytes. The total ion chromatogram (TIC) of the *S. acutum* extract was shown in Figure 1. By extraction of targeted ion and corresponding predicted formula, a total of 52 alkaloid compounds, well separated in time and mass, were observed and assigned with numbers (Table 1). The detailed MS^n^ fragmentation information of each compound was illustrated in Supplementary Materials (Table S1). In order to comprehensively characterize the multi-components in the extract of *S. acutum*, the already reported compounds were collected according to the search of the SCIFINDER, ChemSpider, PubChem, and the literature from PubMed and CNKI. Then, the subgroups, formula, nomenclature, and CAS number (or CID number from PubChem) were sorted. A total of 75 compounds were collected from the aforementioned database. To address the mass spectral analysis for structure characterization, the alkaloids found in the *S. acutum* extracts were grouped into four types in view of their different fragmentation behaviors: (1) morphinans, (2) aporphines, (3) benzylisoquinolines, and (4) protoberberines.

### 2.1. Detection of Morphinan Alkaloids

#### 2.1.1. Fragmentation Analysis of Standard Sinomenine

**Figure 2:**
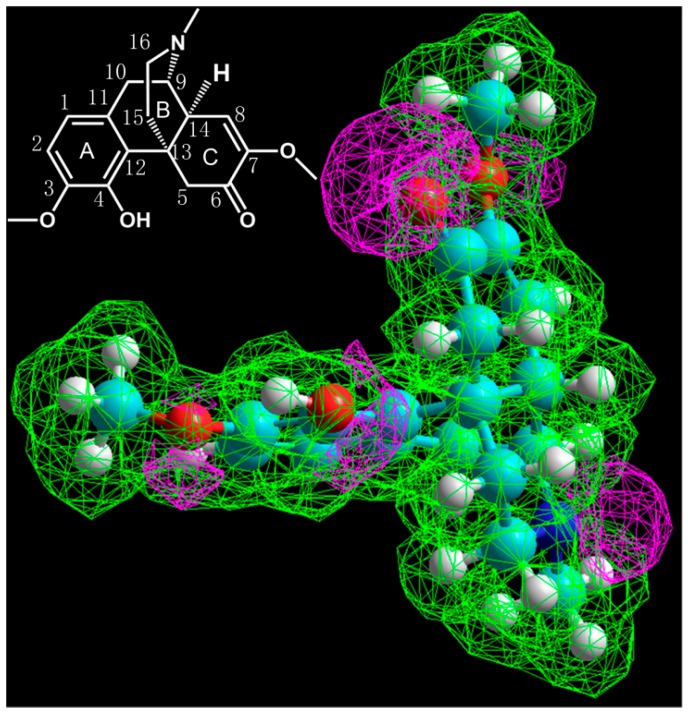
The electrostatic potential plot of the compound model optimized by semi-empirical method, AM1. Purple indicates negative potential while green indicates positive potential. The sites with negative potential were amine group, carbonyl group, hydroxyl group, and ether groups where the initial protonation could happen most likely.

Morphinan alkaloids are the most common alkaloids in *S. acutum*. Although the fragmentation behavior of morphinan alkaloids, including morphine, codeine, and neopinone, have been discussed thoroughly in several previous works [7,11], no potential fragmentation patterns have yet been investigated. Unlike morphine, the morphinan alkaloids in *S. acutum* extract do not have a furan nucleus at C4 and C5, as demonstrated in Figure 2, which cause great differences in MS^n^ fragmentation. The complicated substituents and unsaturated bonds existing in morphinan alkaloids pose great challenges for analysis of their fragmentation patterns.

To facilitate the analysis of morphinan alkaloids in *S. acutum*, the fragmentation of the standard sample of sinomenine was analyzed first. The MS^2^ spectrum of sinomenine appeared more complicated than those of aporphines, benzylisoquinolines, and protoberberines. MS^2^ spectrum of sinomenine displayed abundant MS^2^ products and 7 of them showed high abundance (relative abundance > 50%), while that of other subfamily only displayed one or two product ions (relative abundance > 50%). For a further understanding, MS^3^ information from the target MS^2^ ions was acquired by Orbitrap according to the source injection. TheMS^2^ spectrum and detailed MS^n^ product information were illustrated in Supplementary Materials Figure S1.

**Figure 3:**
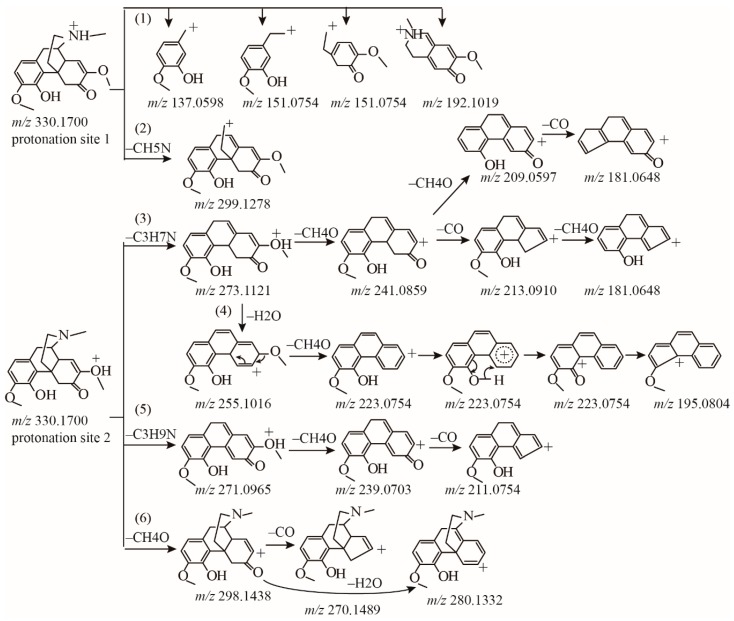
Proposed fragmentation pattern of the protonated sinomenine.

In an attempt to go deep into the fragmentation pattern, the initial protonation sites need to be determined [12,13]. The electrostatic potential plot from simulation suggested that the initial protonation sites should be at an amine group, carbonyl group, hydroxyl group, or two ether groups (Figure 2). Combining the products from MS^n^ spectra, a total of five fragmentation pathways were summarized for sinomenine. First of all, the cleavage of the B ring produces many low mass range ions. Products at *m*/*z* 151.0754, 137.0597, and 192.1019 can be obviously observed. These products from B ring cleavage were considered as pathway (1). Since the protonation of the N, the ion at *m*/*z* 299.1275 was formed by the loss of –CH_5_N (31 Da), as illustrated in the fragmentation pathway (2). This product was only 10% relative abundance. Like other morphine related alkaloids [10], the loss of –C_3_H_7_N (57 Da) was observed at *m*/*z* 273.1116 in the MS^2^ spectrum. Although low abundance was observed, this fragment was critical in the subsequent fragmentation pathway. Most products with high abundances were generated from the ion at *m*/*z* 273.1116. Since the protonation was the oxygen of methoxyl group, the ion at *m*/*z* 273.1116 underwent the loss of methoxyl group (–CH_4_O, 32 Da) at C7 and produced a more stable ion at *m*/*z* 241.0859 corresponding to [C_15_H_13_O_3_]^+^, in which the conjugated π system allowed sharing of the positive charge between multiple atoms with greater stability. Subsequently, the fragment ion at *m*/*z* 213.0910 was formed by the loss of CO from *m*/*z* 241.0859. Interestingly, the ion *m*/*z* 241.0859 also produced the high abundance stable ion *m*/*z* 209.0597. This fragmentation was explained by the loss of CH_4_O from the A ring, supporting the postulated fragmentation pathway of inter-annular charge transfer to produce the benzenium ion. Similar fragmentation was also observed in the transition of *m*/*z* 213.0910 → 181.0648. In addition, a parallel pathway of *m*/*z* 330.1700 → 273.1121 was attributed to the loss of H_2_O. The ion *m*/*z* 273.1116 underwent transformation to form the high abundance *m*/*z* 255.1016 by the loss of H_2_O, and then the loss of the methoxyl group at C7 produced the high abundance ion at *m*/*z* 223.0854. Subsequently, *m*/*z* 223.0854 underwent charge transfer and the loss of CO at C4, yielding the product at *m*/*z* 195.0804. These two parallel fragmentations from *m*/*z* 330.1700 → 273.1121 were defined as pathway (3) and (4). Differently from the reported heroin-related alkaloids, [M + H]^+^ of sinomenine may also lose –C_3_H_9_N (59 Da) and subsequently lose CH_4_O to produce the abundant ion *m*/*z* 239.0703. Similarly with pathway (3) and (4), product at *m*/*z*271.0965 displayed low abundance, while the product from *m*/*z* 271.0965 at *m*/*z* 239.0703 showed high abundance. This fragmentation 330.1700 → 271.0965 → 239.0703 is considered pathway (5). Additionally, the [M + H]^+^ ion of the protonation in methoxyl group directly induced CH_4_O loss to yield ions at *m*/*z* 298.1438. Further loss of CO and H_2_O produced *m*/*z* 280.1332 and 270.1489, respectively. This fragmentation was considered as pathway (6). Most morphinan alkaloids in *S. acutum* extracts contained structure of methoxyl cyclohexenone, may easily loss group of methanol moiety and epoxy bridge. This characteristic fragmentation pattern can indicate the substitution of methoxyl group. All pathways of the standard sinomenine were illustrated in Figure 3.

#### 2.1.2. Detection of Sinomenine Analogues

Similarly with sinomenine, several other types of morphinan alkaloids were identified followed above fragmentation. In the extract ion chromatogram (EIC), *m*/*z* 316.1545 corresponded to *N-*demethyl sinomenine (compound **10**), *m*/*z* 344.1854 corresponded to *N*-methyl sinomenine (compound **9**), and *m*/*z* 657.3134 corresponded to disinomenine (compound **23**) [14]. All these compounds showed fragmentation patterns strikingly similar to that of sinomenine. However, the differences of the product abundances were obviously recorded. Both of [M + H]^+^ ions at *m*/*z* 657.31335 (disinomenine) and *m*/*z* 316.1545 (*N*-demethyl sinomenine), displayed 100% relative abundance of ion at –C_3_H_7_N loss, corresponding to ions at *m*/*z* 600.2596 and 259.0961, respectively, while sinomenine displayed only 22% relative abundance. Followed the aforementioned fragmentation pattern, EIC of *m*/*z* 492.2225 was characterized as sinomenine glucoside (compound **1**), since the obvious 162 Da loss was observed, which indicated the substitution of glucose. Additionally, MS^3^ spectrum from the sinomenine moiety was the same as the MS^2^ spectrum of sinomenine, which exactly suggested the identification of sinomenine glucoside. Another type of morphinan alkaloid, dihydrosinomenine, also exhibited a similar fragmentation pattern to that of sinomenine. Only the differences were observed in abundance of the MS^2^ products. In EIC, *m*/*z* 318.1699 corresponded to *N*-demethyl dihydrosinomenine (compound **6**). The products from pathway (4) at *m*/*z* 225.0907 and 257.1168 were only of ~10% relative abundance, while that of sinomenine at *m*/*z* 223.0751 and 255.1013 were of about ~95% relative abundance. In the case of *m*/*z* 332.1854, which corresponded to dihydrosinomenine (compound **11**) displayed the same fragmentation pattern with sinomenine, and also showed low abundance of products from pathway (4).

**Figure 4:**
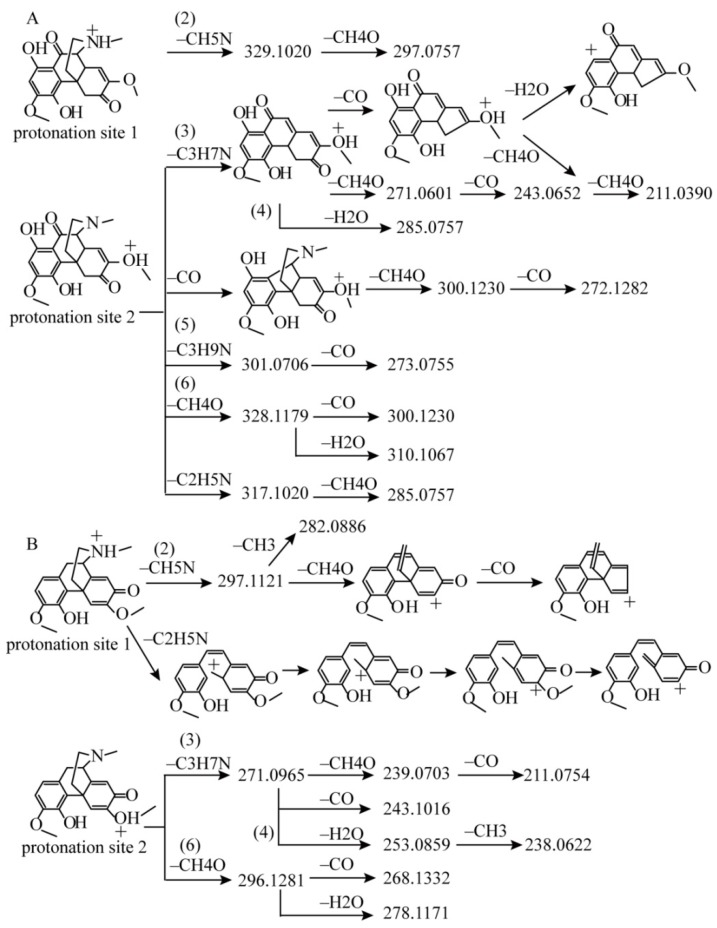
Proposed fragmentation pattern of the protonated (**A**) 1-hydroxy-10-oxo-sinomenine and (**B**) protonated sinoacutine.

In addition to the aforementioned compounds, two more compounds should be noteworthy. One corresponded to *m*/*z* 360.14429 in EIC (compound **20**). The products from pathway (1) were not recorded, probably because of their low abundance in the background. Common products from pathways (2), (3), (4), and (5) were all displayed in the MS^2^ spectrum, as illustrated in Figure 4A. Compound **20** is speculated to have the same skeleton as that of sinomenine. The predicted formula based on the accurate mass suggested additional substituted hydroxyl and carbonyl groups in skeleton as compared to sinomenine. Through database searching and literatures, this compound was tentatively identified as 1-hydroxy-10-oxo-sinomenine [15,16]. Most fragmentations of this compound were similar to those of sinomenine, with two exceptions. First, the carbonyl group at C10 may lose readily. The product at *m*/*z* 275.0914, resulting from the direct loss of CO to produce the transition from *m*/*z* 360.1442 → 303.0863, displayed the highest abundance in the MS^2^ spectrum. Additionally, products from CO loss at *m*/*z* 332.1492 (~50% relative abundance), 300.1230 (~30% relative abundance), and 272.1282 (~40% relative abundance) were predominantly observed in MS^2^ spectrum. For sinomenin, the product from directly CO loss was only 6.5%. Thus, this pathway was typical for C10 carbonyl morphinan alkaloids. Second, compound **20** exhibited the product from C_2_H_5_N loss at *m*/*z* 317.1020, which was absent of the sinomenin. The fragmentation pattern was demonstrated in Figure 4A.

The second noteworthy compound corresponded to *m*/*z* 328.1543 in the EIC (compound **34**) with predicted formula as C_19_H_22_O_4_N. It was grouped into morphinan alkaloids containing substructure of cyclohexadienone, according to the degree of unsaturation. The [M + H]^+^ ion underwent a fragmentation pathway similar to pathways (2), (3), (4), and (6). The ions from pathway (5) were absent because of the present of cyclohexadienonein C-ring. The inherent cyclohexadienone in C-ring could enhance the π-conjugation of the products. Thus, the ions from pathway (2) were present in high abundance as compared to those from sinomenine. The ion at *m*/*z* 297.1117 was about 50% relative abundance, and subsequently yielding the *m*/*z* 265.0856 from the CH_4_O loss displayed 100% relative abundance. This compound was finally identified as sinoacutine [17]. The fragmentation pattern was illustrated in Figure 4B.

Compound **33** was later appended based on further analysis. Identical products with sinomenine were produced from the [M + H]^+^ ion at *m*/*z* 330.1700. However, the base peak was observed at *m*/*z* 298.14386, while the others were all at low abundance (<30% base peak). For characterizing the structure in detail, an elaborate MS^2^ experiment was performed. Products corresponding to *m*/*z* 209.0595, 213.0907, 223.0752, and 241.0857 confirmed that the fragmentation pathway was the same as that for sinomenine. Compound **33** was finally identified as isosinomenine [18]. The MS^2^ spectrum and pathway were shown in Supplementary Materials Figure S1.

**Figure 5:**
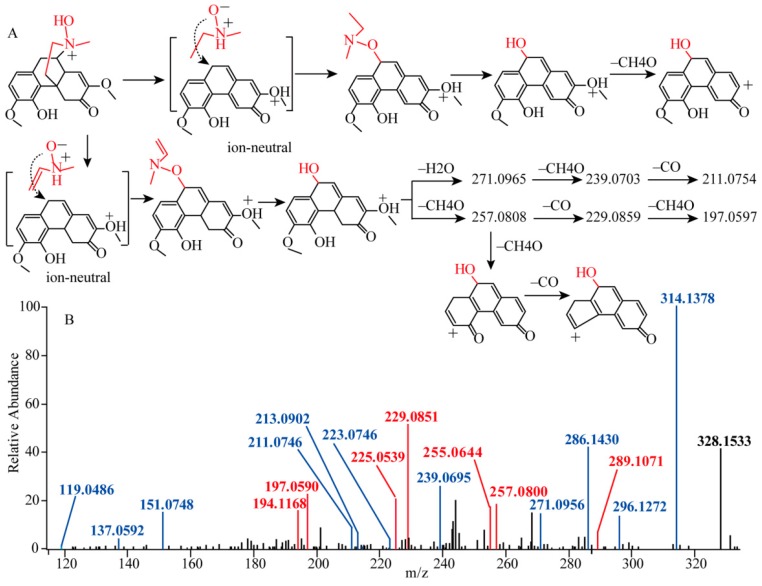
(**A**) the fragmentation pathway of the produced ion-neutral complex intermediate, (**B**) the MS^2^ spectrum of the sinomenine *N*-oxide, the ions in blue suggested the same fragments with sinomenine, the ions in red suggested the special fragments generated from the ion-neutral complex intermediate.

Compound **2** provided the [M + H]^+^ ion at *m*/*z* 346.1650. The predicted formula based on the accurate mass suggested that an additional hydroxyl group was substituted, compared to sinomenine. The product [M + H − H_2_O]^+^ was also predominantly displayed in the MS^2^ spectrum, confirming additional hydroxyl substitution. The CID of [M + H]^+^ strikingly produced ions at *m*/*z* 314.1385, 296.1279, and 286.1437, mainly via pathway (6). Detailed investigation of the low-abundance products indicate that ions produced via pathways (1), (2), (3), (4), and (5) were all observed in the MS^2^ spectrum (Figure 5A). As illustrated in Figure 5B marked in green, these ions included *m*/*z* 137.0592 and 151.0748 from pathway (1), *m*/*z* 299.1279 from pattern (2), *m*/*z* 213.0602 from pattern (3), *m*/*z* 223.0754 from pathway (4), *m*/*z* 271.0956, 239.0695, and *m*/*z* 211.0746 from the pattern (5). Elaborate investigation of the MS^3^ experiment confirmed that the same fragmentation of these products occurred in the case of sinomenine (Supplementary Materials Table S1). Finally, compound **2** was potentially characterized as sinomenine *N*-oxide. Of interest, in the MS^2^ spectrum, the product ions at *m*/*z* 197.0599 ([C_13_H_9_O_2_]^+^), 225.0539 ([C_14_H_9_O_3_]^+^), 229.0856 ([C_14_H_13_O_3_]^+^), 255.0644 ([C_15_H_11_O_4_]^+^), 257.0804 ([C_15_H_13_O_4_]^+^), and 289.1071 ([C_16_H_17_O_5_]^+^) were found in relatively high abundance in the middle mass range. The predicted formulas of these four products each contain one more oxygen atom as compared to those of sinomenine fragments at *m*/*z* 181.0648 ([C_13_H_9_O]^+^), 209.0597 ([C_14_H_9_O_2_]^+^), 213.0910 ([C_14_H_13_O_2_]^+^), 239.0703 ([C_15_H_11_O_3_]^+^), 241.0859 ([C_15_H_13_O_3_]^+^), and 273.1121 ([C_16_H_17_O_4_]^+^). Many reports have established that the production of complex intermediates or cation transfer is involved in fragmentation [3,19,20]. Our findings implied that an anion-neutral complex intermediate might be involved in the sinomenine nitrogen oxide fragmentation process. The potential fragmentation pathway was shown in Figure 5. The detailed MS^n^ spectra were illustrated in Supplementary Materials Figure S2.

### 2.2. Detection of Aporphines and Benzylisoquinolines

Several studies have elucidated the detailed fragmentation patterns of aporphine and benzylisoquinoline alkaloids [4,21]. In studying the MS^2^ spectraof aporphine and benzylisoquinoline alkaloids, the fragment ions [M + H − 45]^+^, [M + H − 31]^+^, and [M + H − 17]^+^ were always observed initially depending on the type of nitrogen substitution. Thereafter, the neutral losses of CH_3_ or CH_4_O were observed in aporphine alkaloids. A total of 11 compounds followed this fragmentation pattern. Eight of them were structurally identified. Another four peaks lost 45 Da with prominent abundance in the MS^2^ spectrum, however, no identification was provided because of the puzzling formulae predicted from the accurate mass measurements, and no hits can be accessed from the database search.

For benzylisoquinoline alkaloids, in agreement with the literature [4], the product resulting from the loss of the characteristic NHR_1_R_2_ (where R_1_ and R_2_ represent the substituent groups for the nitrogen atom) was predominant in the MS^2^ spectrum. Then, the characteristic ions from α- and β-cleavages were produced. Although a similar fragmentation pattern was provided, the intensity patterns look remarkably different. For compound **16**, quasi-molecular ion [M + H]^+^ produced a high intensity product at *m*/*z*192.1015 and a low intensity ion at *m*/*z* 285.1115 (–CH_5_N) in the MS^2^ spectrum. Further MS^3^ spectrum from *m*/*z* 285.1115 showed the similar fragmentation pattern with other benzylisoquinoline alkaloids. Comparison of the MS^2^spectraof compounds **16** and **37** (magnocurarine) was provided in Figure S3. Although both of the compounds were assigned to the benzylisoquinoline alkaloids and produced similar fragmentation patterns, there was a significant difference in intensity of the MS^2^ spectrum. This compound was finally identified as 3′-hydroxy-*N*-methylcoclaurine. Correspondingly, compound **13** was characterized as 3′-hydroxy-*N*-methylcoclaurine glucoside.

### 2.3. Detection of Protoberberines

To investigate of the protoberberines, several standards of the protoberberines, including tetrahydroberberine, tetrahydropalmatine, berberrubine, jatrorrhizine, columbamin, palmatine, and berberine were analyzed to analyze their potential fragmentation pattern. The MS^n^ information was illustrated in Supplementary Materials Tables S1 and S2.In agreement with previous studies [6,22], protoberberine alkaloids with one methoxy group at C9 or C10 generated single ions at [M − •CH_3_]^+•^ in the MS^2^ spectrum, such as berberrubine, while protoberberine alkaloids with two methoxy groups at C9 and C10 generated ions at [M − •CH_3_ − •H]^+^ and [M − •CH_3_]^+•^ in the MS^2^ spectrum, including berberine, jatrorrhizine, columbamin and palmatine. In addition, the [M − •CH_3_ − •H − CO]^+^ ion was expected to show high abundance in the MS^3^ spectrum. Based on the above regular patterns, compounds **47**, **48**, **49**, **51**, and **52** were structurally identified. In the case of compound **50**, since the literature suggested that palmatine and pseudo-palmatine had different base peaks in the MS^2^ spectrum [6,23],compound **50** was potentially identified as pseudo-berberine, correspondingly.

Two additional compounds produced different MS^n^ spectra. For compound **43**, its [M + H]^+^ ion produced a single [M − •CH_3_]^+•^ ion in the MS^2^ spectrum. The [M − •CH_3_]^+•^ ion further produced [M − •CH_3_ − •CH_3_]^+^ in the MS^3^ spectrum, rather than the usual ions of [M − •CH_3_ − •H − CO]^+^ and [M − •CH_3_ − •H]^+^. This compound was identified as stepharanin [24]. As for compound **41**, its [M + H]^+^ ion produced [M + H − •CH_3_]^+•^ in the MS^2^ spectrum, and it further fragmented into the ion [M + H − •CH_3_ − •CH_3_]^+^ in the MS^3^ spectrum. Reports on the fragments of 8-oxoprotoberberine alkaloids have differed. Phuong et al. and Phengxay et al. demonstrated that 8-oxoprotoberberine produced [M + H − •CH_3_]^+•^ and [M + H − •CH_3_ − •H − CO]^+^ in the MS^2^ spectrum, and then [M + H − •CH_3_]^+•^ produced [M + H − •CH_3_ − •CH_3_]^+^ and [M + H − •CH_3_ − •CH_3_−CO]^+^ in the MS^3^ spectrum by means of the hybrid ion-trap MS [6,20,21]. In this work, compound **41** was identified as oxypalmatine, as oxypalmatine has been reported in *S. acutum* [16]*.*

### 2.4. Other Alkaloids

Compound **15** presented three ionization types in the MS^1^ spectrum, namely, [M + H]^+^ at *m*/*z* 398.1370, [M + Na]^+^ at *m*/*z* 420.1183, and [M+K]^+^ at *m*/*z* 436.0920. The isotopic distribution and accurate mass measurement suggested that compound **15** contained the element chlorine. The MS^2^ spectrum featured a high-abundance product ion at *m*/*z* 341.0783, corresponding to the neutral loss of C_3_H_7_N. A search in the database and literature [16] revealed that this compound was identified as acutumine [25]. Based on this structural characterization, the fragmentation pattern was deduced. In the MS^2^ spectrum, [M + H − C_3_H_7_N]^+^ was unexpectedly observed as the base peak, mainly because the quaternary carbocation conjugating with two π bonds was more stable. Then, the neutral loss of H_2_O occurred to produce *m*/*z* 323.0681. Additionally, the loss of HCl from *m*/*z* 341.0786 was also observed and produced the product at *m*/*z* 287.0914. The formation of the ion at *m*/*z* 309.0524 from *m*/*z* 341.0786 was the same as that in pathway (3) of sinomenine (Supplementary Materials Figure S4). Following the similar fragmentation pattern, compound **8** was identified as acutumidine.

For compound **24** with [M + H]^+^ at *m*/*z* 360.18024, analysis could not be conducted as the MS^2^ spectrum since it appeared less information. Product ions at [M + H − C_3_H_7_N]^+^ and [M + H − C_3_H_7_N − H_2_O]^+^ were predominant in the MS^2^ spectrum. Interestingly, this distribution of the MS^2^ spectrum was extremely similar to the distributions of the spectra of acutumine and acutumidine. These observations strongly suggested that the quaternary carbocation of the ion at [M + H − C_3_H_7_N]^+^ was generated. By searching the database, this compound was identified as cephatonine. Correspondingly, compound **4** was identified as cephatonineglucoside. For this compound, except for the high abundance products from aglycone, [M + H]^+^ might directly produce [M + H − C_2_H_4_N]^+^ (*m*/*z* 479.1910), [M + H − C_3_H_7_N]^+^ (*m*/*z* 465.1755),and [M + H − C_3_H_7_N − H_2_O]^+^ (*m*/*z* 447.1650). 

By combining the database search and partial fragment matching, we potentially characterized two additional compounds that did not belong to any subgroups [16]. One was compound **17**. It gave the pseudo-molecular ion at *m*/*z* 314.1394. In the MS^2^ spectrum, the base peak was at *m*/*z* 299.1155, suggesting methoxy substitution, and the fragment at *m*/*z* 151.0751 ([C_9_H_11_O_2_]^+^) and 137.0594 ([C_8_H_9_O_2_]^+^) suggested the presence of the methoxy-phenol unit. These important fragments allowed this compound to be identified as feruloyltyramine. The second was compound **26**. It gave the pseudo-molecular ion at *m*/*z* 298.1442. In the MS^2^ spectrum, ions at *m*/*z* 192.1018, 269.1173, and 281.1174 were observed. Through database search and fragment matching, this compound was identified as stepharine [26].

## 3. Materials and Methods

### 3.1. Materials and Chemicals

*S. acutum* was purchased from Anhui FengyuanTongling Chinese Herbal Medicine Co., Ltd. (Anhui, China). Standards of higenamine, sinomenine, magnoflorine, tetrahydroberberine, tetrahydropalmatine, berberrubine, jatrorrhizine, columbamine, berberine, and palmatine were purchased from the Shanghai Yuanye Biological Technology Co., Ltd. (Shanghai, China). All aqueous solutions were prepared with bottled pure water (Wahaha, Hangzhou, China). High performance liquid chromatography grade acetonitrile and methanol were purchased from Fisher Scientific (Fair Lawn, NJ, USA). Other chemicals and solvents were all of analytical grade.

### 3.2. Sample Preparation

Therattan stem of *S. acutum* was authenticated according to its morphological characteristics by the Professor Shengjin Liu in Nanjing University of Chinese Medicine. The raw material of *S. acutum* (2.0 g) was ultrasonicated twice for 120 min in 20 mL ethanol–water (75:25, volume ratio). The two extracts were mixed, filtered, and then evaporated to dryness under vacuum. The residue was redissolved in 20 mL water and vortexed for 10 min. Centrifugation at 18,000 rpm for 10 min provided a 200 μL aliquot which was transferred to a vial.

### 3.3. Chromatographic Separation Conditions

Chromatographic separation was performed using a Dionex U3000 Ultra Performance Liquid Chromatography (UPLC) system with a Phenomenex Kinetex C18 (2.1 mm × 100 mm, 2.6 μm) column with the column temperature set at 40 °C. The mobile phase consisted of solvent A (0.1% formic acid in water) and solvent B (acetonitrile). The total run time was 29 min including five linear gradient components: from 0 to 2 min, 5% B; 2 to 20 min, 5–25% B; 20 to 22 min, 25–75% B; 22 to 25 min, 75–5% B; 25 to 29 min, 5% B. The flow rate was 0.25 mL/min. Each time, a 5 μL aliquot was used for analysis. 

### 3.4. MS Conditions

MS experiments were performed with a LTQ Orbitrap linear ion trap hybrid MS (Thermo Fisher Scientific, Inc., Bremen, Germany) equipped with an electrospray ionization (ESI) source. The positive ESI mode was used with the spray voltage of 3.5 kV. The temperature of the heated capillary was 300 °C. The temperature of the ESI probe was 350 °C. The flows of sheath gas and auxiliary gas were set as 40 units and 15 units, respectively. The scan range of *m*/*z* was 200–800 with the Orbitrap analyzer. MS^n^ data were acquired in the data–dependent acquisition mode. Target ions selected for fragmentation were obtained by dynamic exclusion for 20 s. The normalized collision energy values for both MS^2^ and MS^3^ were 35, and the ion selection thresholds were 10,000 and 1000 counts, respectively. To elaborately investigate the fragmentation pattern, the standard of sinomenine was analyzed by source injection. We first set out to monitor the MS^2^ spectrum from the quasi-molecular ion of standard sinomenine. On the basis of MS^2^ spectrum, further MS^3^ information was acquired from the targeted products with high relative abundance.

Instrument control, data acquisition, and data analysis were performed using Xcalibur 2.2 SP11.48 (Thermo Fisher Scientific, Inc., Bremen, Germany) software.

### 3.5. Simulation

The theoretical calculations of sinomenine were carried out by HyperChem (Hyper, Gainesville, FL, USA). The semi-empirical method, AM1, was used to calculate the formal charges and optimize the geometry. The molecular orbitals were generated by these optimized models. The three-dimensional iso-surface of electrostatic potential was plotted.

## 4. Conclusions

Significant progress has been made in the MS characterization of alkaloids. In this study, we analyzed the alkaloids of the morphinan compounds, aporphines, protoberberines, and benzylisoquinolines in the *S. acutum* extract; they exhibited some similarities but also considerable differences, especially in the case of protonated morphinan compounds. Loss of the methanol moiety and epoxy bridge were always observed in the MS^2^ spectrum. Although their fragmentation patterns were similar, the intensity patterns appeared remarkably different. In general, the present study contributes by proposing some universal fragmentation rules for addressing the structural characterization of alkaloids in complex herbal medicines.

## Figures and Tables

**Table 1:** Retention time, accurate mass data, and identification of alkaloids from the *S.acutum.*

No.	t*_R_* (min)	*m*/*z*	Formula ([M + H]^+^)	ppm	Identification
1	1.97	492.2225	C_25_H_34_O_9_N	−0.308	Sinomenine glucoside
2	2.00	346.1650	C_19_H_24_O_5_N	0.111	Sinomenine *N*-oxide
3	2.05	434.1818	C_22_H_28_O_8_N	0.817	Higenamine glucoside
4	2.74	522.2331	C_26_H_36_O_10_N	−0.283	Cephatonine glucoside
5	3.21	346.1643	C_19_H_24_O_5_N	−1.789	Unknown
6	3.76	318.1699	C_18_H_24_O_4_N	−0.065	*N*-demethyl dihydrosinomenine
7 ^1^	4.48	272.1285	C_16_H_18_O_3_N	0.330	Higenamine
8	4.88	384.1209	C_18_H_23_O_6_NCl	0.256	Acutumidine
9	4.93	344.1854	C_20_H_26_O_4_N	−0.215	*N*-methyl sinomenine
10	4.99	316.1545	C_18_H_22_O_4_N	0.145	*N*-demethyl sinomenine
11	5.59	332.1854	C_19_H_26_O_4_N	−0.827	Dihydrosinomenine
12	5.69	448.1973	C_23_H_30_O_8_N	0.707	Coclaurine glucoside
13	5.78	478.2079	C_24_H_32_O_9_N	1.656	3′-hydroxy-*N*-methyl coclaurine glucoside
14 ^1^	6.61	330.1700	C_19_H_24_O_4_N	0.167	Sinomenine
15	6.74	398.1362	C_19_H_25_O_6_NCl	−0.657	Acutumine
16	7.64	316.1543	C_18_H_22_O_4_N	−0.110	3′-hydroxy-*N*-methylcoclaurine
17	7.94	314.1394	C_18_H_20_O_4_N	2.182	Feruloyltyramine
18	8.62	476.2283	C_25_H_34_O_8_N	0.407	Magnocurarine4′-*O*-glucopyranoside
19	8.92	358.1646	C_20_H_24_O_5_N	−0.319	Hydroxyl magnoflorine
20	9.23	360.1438	C_19_H_22_O_6_N	−0.334	1-hydroxy-10-oxo-sinomenine
21	9.25	358.1646	C_20_H_24_O_5_N	−0.319	Hydroxyl magnoflorine
22	9.56	286.1442	C_17_H_20_O_3_N	0.400	Coclaurine
23	9.57	657.3165	C_38_H_45_O_8_N_2_	−0.780	Disinomenine
24	9.69	360.1802	C_20_H_26_O_5_N	−0.309	Cephatonine
25	9.79	300.1595	C_18_H_22_O_3_N	0.030	*N*-methylcoclaurine
26	9.86	298.1442	C_18_H_20_O_3_N	1.442	Stepharine
27	9.98	358.1647	C_20_H_24_O_5_N	−0.229	Hydroxyl magnoflorine
28	10.47	344.1852	C_20_H_26_O_4_N	−1.147	Tembetarine
29	10.56	328.1547	C_19_H_22_O_4_N	0.325	Unknown
30	10.70	370.1646	C_21_H_24_O_5_N	−0.863	Unknown
31 ^1^	10.91	342.1698	C_20_H_24_O_4_N	−0.185	Magnoflorine
32	11.16	328.1545	C_19_H_22_O_4_N	0.145	Unknown
33	11.52	330.1699	C_19_H_24_O_4_N	−0.196	Isosinomenine
34	11.59	328.1547	C_19_H_22_O_4_N	0.991	Sinoacutine
35	11.85	360.1437	C_19_H_22_O_6_N	−1.177	Unknown
36	11.96	340.1543	C_20_H_22_O_4_N	−0.102	*N*-Methyl bulbocapnine
37	12.19	314.1753	C_19_H_24_O_3_N	0.795	Magnocurarine
38	12.44	342.1698	C_20_H_24_O_4_N	−0.54	Laurifoline
39	13.98	356.1856	C_21_H_26_O_4_N	−0.238	*N*-methyl isocorydine
40	14.62	390.1548	C_20_H_24_O_7_N	−0.406	Unknown
41	14.67	368.1492	C_21_H_22_O_5_N	−0.215	Oxypalmatine
42	15.34	322.1078	C_19_H_16_O_4_N	1.166	Menisporphine
43	15.43	324.1231	C_19_H_18_O_4_N	0.048	Stepharanine
44	15.74	322.1076	C_19_H_16_O_4_N	0.793	Menisporphine
45	15.77	356.1854	C_21_H_26_O_4_N	−0.603	*N*-methyl corydine
46	16.15	374.1596	C_20_H_24_O_6_N	−0.625	Unknown
47	17.77	338.1386	C_20_H_20_O_4_N	−0.221	Dehydrocorydalmine or its isomer
48	18.15	338.1387	C_20_H_20_O_4_N	−0.043	Dehydrocorydalmine or its isomer
49 ^1^	18.42	338.1384	C_20_H_20_O_4_N	−0.842	Jatrorrhizine
50	18.42	336.1231	C_20_H_18_O_4_N	0.314	Pseudoberberine
51 ^1^	18.74	338.1383	C_20_H_20_O_4_N	−1.108	Columbamin
52 ^1^	21.38	352.1543	C_21_H_22_O_4_N	−0.098	Palmatine

^1^ Confirmed by standards.

## Supplementary Materials

The following are available online, Figure S1: The MS^n^ spectra of the(**A**–**E**) sinomenine and (**F**–**K**) isosinomenine, Figure S2: The MS^n^ spectra of the sinomenine *N*-oxide, Figure S3: The comparison between the magnocurarine and 3′-hydroxy-*N*-methylcoclaurine, (**A**,**B**) the MS^n^ spectra of themagnocurarine, and (**C**–**E**) 3′-hydroxy-*N*-methylcoclaurine. Figure S4: The proposed MS fragmentation pattern of the acutumine, Table S1: MS^n^ data in positive mode of compound observed in the *S. acutum*, Table S2: MS^n^ information of the standards.
