# Supplementary material for: Liquid Chromatography Coupled with Linear Ion Trap Hybrid OrbitrapMass Spectrometry for Determination of Alkaloids in Sinomeniumacutum
Source: Molecules. 2018 Jul 4;23(7):1634. doi: 10.3390/molecules23071634 (PMC6099952; doi:10.3390/molecules23071634)
Supplement: Supplementary file 1 [file molecules-23-01634-s001.pdf]

molecules

MDPI

Supplemental Materials

Liquid chromatography coupled with linear ion trap hybridOrbitrap mass spectrometry method for determination of alkaloids in the *Sinomeniumacutum*

Jinjun Shan[1,#], Xia Zhao[1,#],CunsiShen[1], JianjianJi[1], Jianya Xu[1], ShouchuanWang[1],Tong Xie[1,*], WenjunTong[2,*]

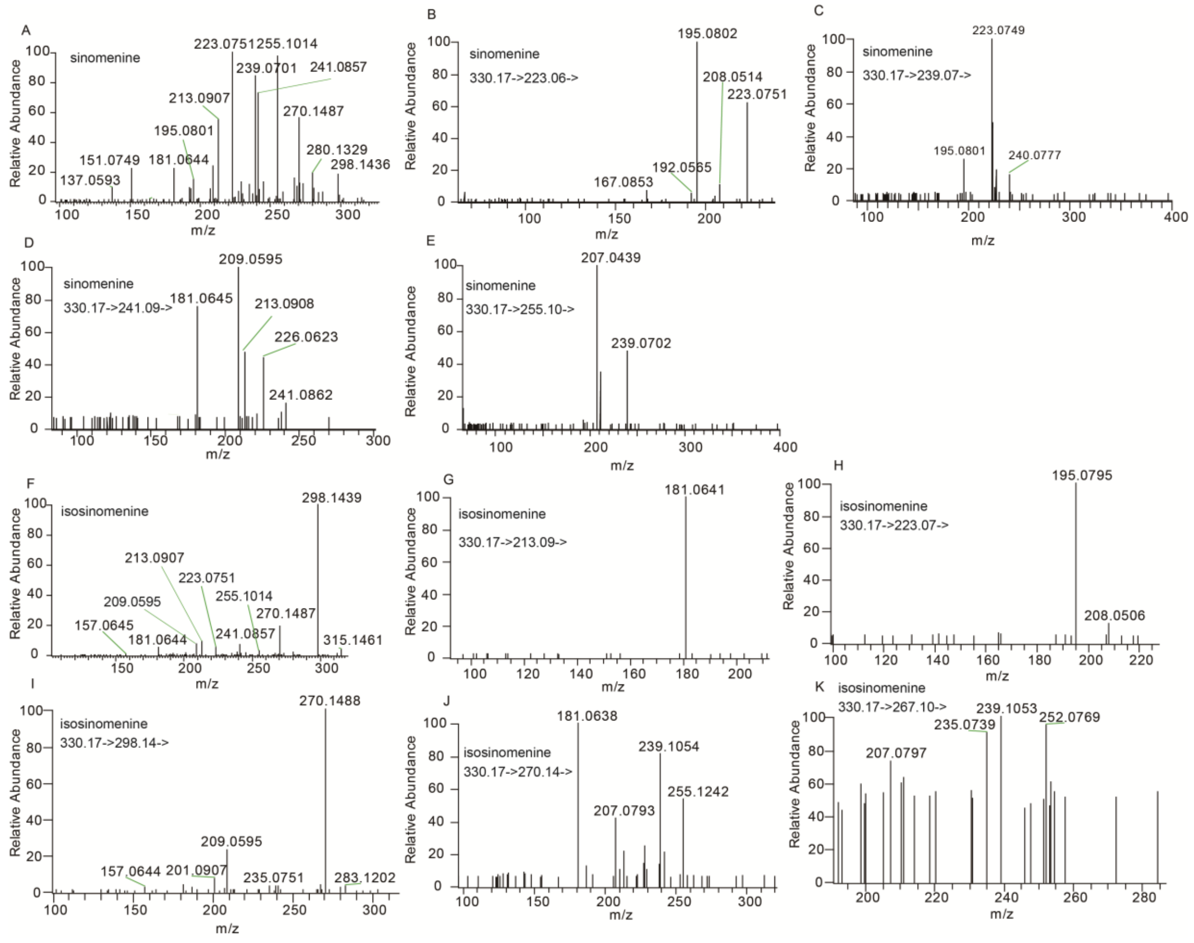

Figure S1. The MS^n spectra of the (**A**-**E**) sinomenine and (**F**-**K**) isosinomenine.

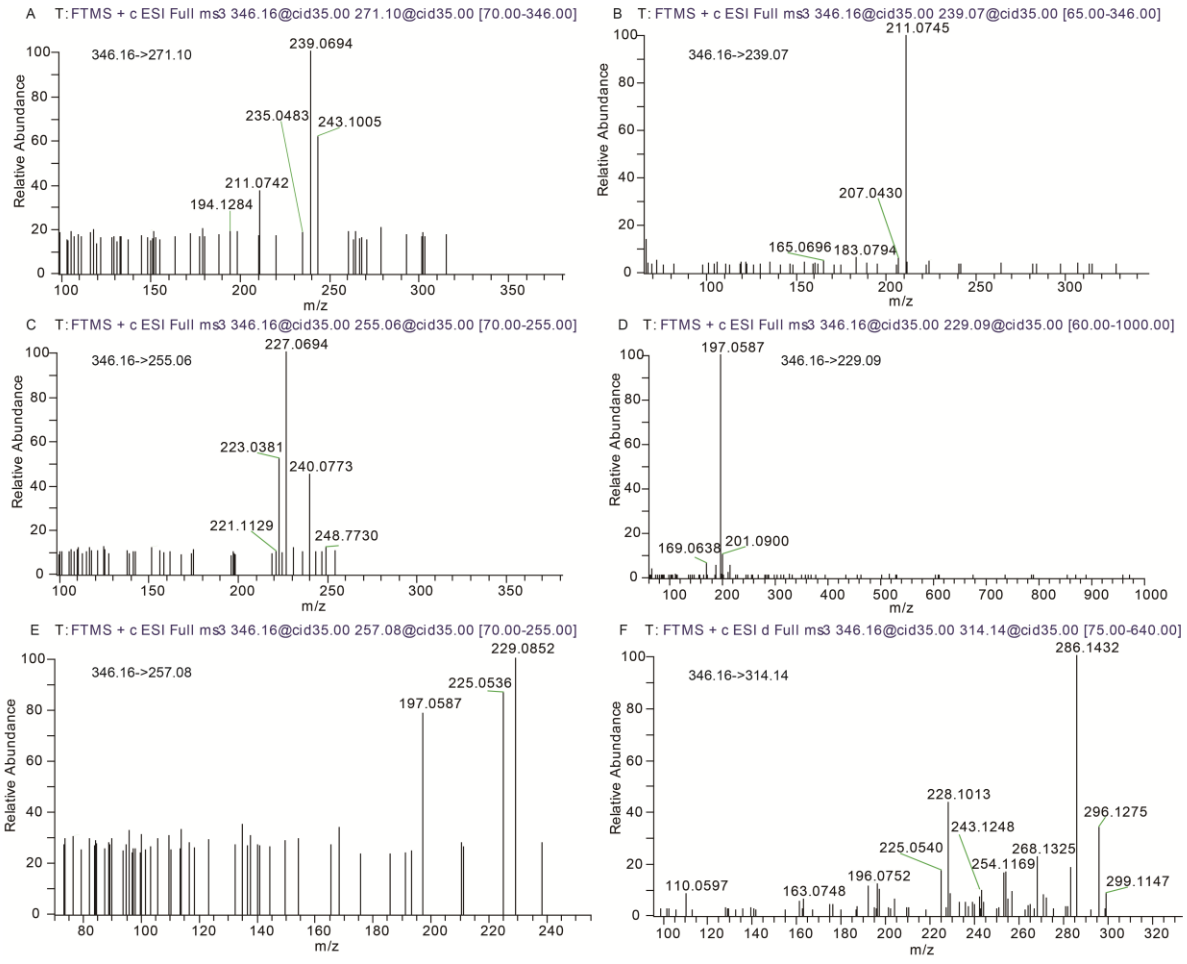

Figure S2. The MS^n spectra of the sinomenine N-oxide.

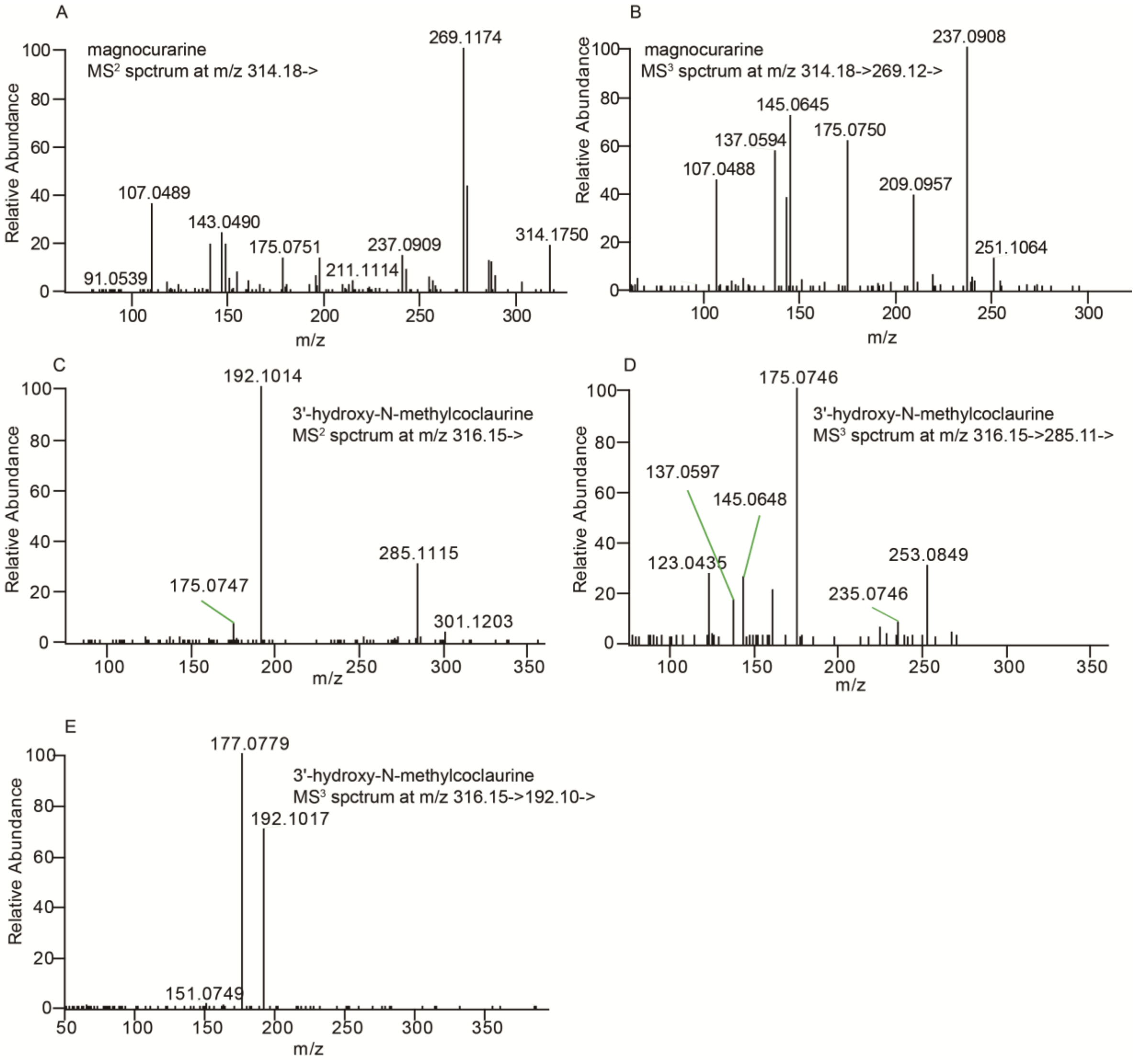

Figure S3. The comparison between the magnocurarine and 3'-hydroxy-N-methylcoclaurine, (**A**-**B**) the MS^n spectra of the magnocurarine, and (**C**-**E**) 3'-hydroxy-N-methyl coclaurine.

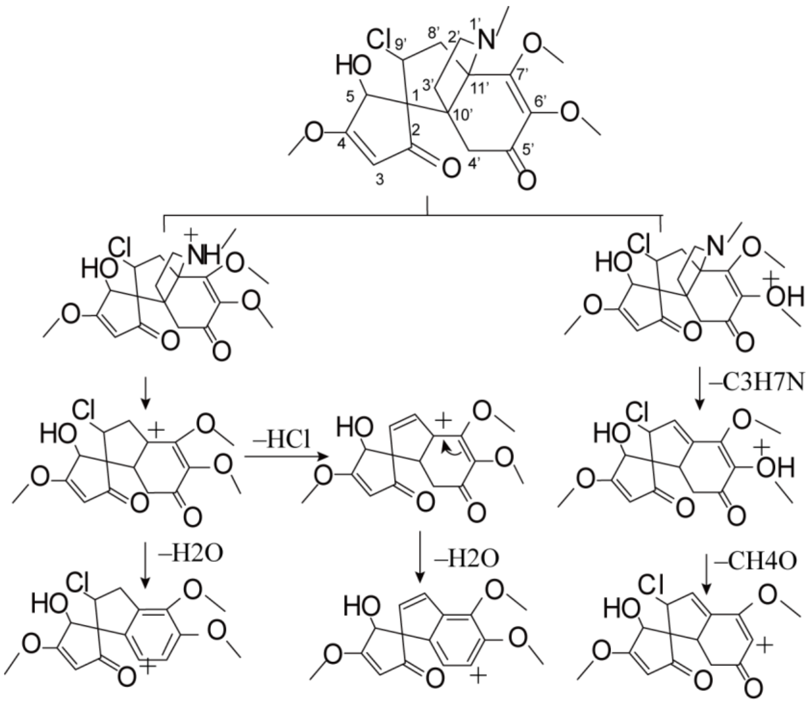

Figure S4. The proposed MS fragmentation pattern of the acutumine.

Table S1. MS^n data in positive mode of compound observed in the *Sinomeniumacutum*.

No.	MS^{2}data	MS^3 data
1	223.0751, 241.0858, 255.1015, 267.1015, 273.1121, ***287.1278,*** 299.1278, 330.1699, 417.1545, 435.1650, 449.1807, 460.1966	223.0750, 255.1014
2	137.0591, 151.0747, 178.0856, 181.0644, 190.0855, 194.1168, 195.1015, 195.0801, 197.0589, 209.0594, 211.0746, 213.0902, 223.0751, 225.0539, 229.0851, 239.0695, 241.0855, 243.1246, 244.1324, 253.1012, 255.0644, 257.0800, 268.1324, 271.0956, 286.1429,289.1066, 296.1272, 314.1377, 328.1532	[***229.0851***] 197.0589; [***239.0695***] 183.0796, 207.0432, 211.0745; [***255.0644***] 223.0385, 227.0694, 240.0772; [***257.0800***] 197.0590, 225.0539;[***271.0956***] 211.0744, 239.0695, 243.1005; [***314.1383***] 178.0857, 268.1326, 286.1431, 296.1274;
3	255.1014***, 272.1282***	107.0487, 123.0437, 136.0753, 143.0488, 145.0644, 161.0592, 255.1010
4	243.1014, 271.0964, ***285.1122,*** 303.1227, 317.1381, 447.1648, 465.1752, 479.1908, 490.2066, 504.2224	254.0935, 270.0884
5	257.0803, ***328.1541***	191.0696, 239.0699, 268.1328, 286.1432, 296.1277
6	137.0594, 157.0644, 183.0800, 193.0644, 201.0905, 211.0749, 215.1062, ***243.1010,*** 269.1166, 300.1587	137.0595, 155.0851, 161.0593, 183.0800, 193.0643, 201.0905, 211.0749, 215.1061, 228.0776
7	107.0488, 123.0438, 161.0894,***255.1010***	107.0489, 123.0438, 145.0645, 161.0595
8	299.0915, 305.1205, 309.0525, 316.1180, ***341.0780,*** 348.1442, 352.0944, 367.0941	255.0652, 273.0757, 287.0916, 305.1021, 323.0682
9	181.0644, 193.0644, 195.0800, 209.0594, 213.0907, 223.0751, 239.0701, 241.0855,***255.1014***, 267.1012, 271.0963, 273.1117, 281.1168, 299.1273, 326.1382	195.0799, 223.0751, 227.1060, 240.0774
10	137.0595, 151.0752, 178.0859, 209.0594, 213.0907, 223.0750, 227.0701, ***239.0699,*** 241.0854, 255.1012, 256.1328, 259.0960, 267.1011, 269.1170, 271.0960, 273.1118, 287.1273, 299.1273	193.0643, 207.0436, 211.0748
11	137.0594, 165.0694, 181.0644, 183.0801, 187.0751, 193.0645, 195.0801, 201.0907, 211.0751, ***213.0907,*** 215.1062, 225.0906, 227.1065, 229.1095, 243.1012, 251.1063, 257.1168, 269.1171, 275.1279, 283.1329, 300.1593, 314.1750	153.0694, 181.0644, 185.0957, 198.0671
12	269.1174, ***286.1438***	107.0487, 151.0750, 175.075, 269.1172
13	77.0781, ***192.1020,*** 316.1540	151.0749, 177.0779
14	137.0594, 151.0749, 181.0643, 192.1017, 193.1095, 195.0802, 207.0802, 209.0593, 213.0906, 223.0751, 239.0700, 241.0856, 255.1013, 259.0964, 267.1013, 270.1486, 271.0963, 273.1120, 280.1326, 287.1277, 298.1436	**[*223.0751*]**195.0802, 208.0514, 223.0750; **[*239.0700*]**207.0439, 211.0753, 239.0702; **[*241.0856*]**181.0645, 209.0594, 213.0908, 226.0623; **[*255.1013*]** 195.0801, 223.0749, 227.1059, 240.0780; **[*270.1486*]**195.0800, 227.1063, 255.1252
15	305.1021, ***341.0786,*** 362.1597, 366.1100	287.0915, 305.1021, 323.0681
16	175.0748, 192.1017, 285.1118	**[*192.1017*]**177.0779, 192.1016; **[*285.1118*]**115.0536, 123.0434, 137.0590, 143.0485,

		161.05906, 175.0745, 253.0850
17	137.0594, 151.075,***299.1155,*** 314.1385	252.1017, 280.0972, 283.0839
18	***269.1172,***314.1749	107.0487, 269.1169, 299.1513
19	225.0907, 253.0857, 281.0808, ***313.1070***	225.0905, 253.0857, 281.0808, 298.0834
20	137.0595, 151.0751, 167.0336, 192.1014, 211.0385, 243.0648, 257.0804, 258.1123, 269.0808, 271.0600, 275.0910,285.1116, 297.0756, 300.1226, 303.1221, 311.0908, 317.1377, 328.1178, 332.1485, 342.1328, 345.1202	**[*275.0910*]**211.0385, 243.0651, 247.0958, 257.0803; [***328.1178***] 272.1272, 296.1274, 300.1222
21	253.0857, 285.1121, 295.0963,***313.1071***, 328.1540, 340.1540	223.0594, 253.0858, 281.0808, 285.0756
22	107.0488, 137.0594, 145.0644, 151.0750, 163.0750, 175.0750,178.0858, ***269.1174***, 286.1437	107.0488, 137.0594, 143.0489, 145.0645, 175.0749, 209.9573, 237.0908
23	450.1633, 495.2211, 509.1597, 511.1752, 514.2222, 517.2333, 526.2223, 540.2380, 557.2647, 566.2174, 568.2332, 582.2490, 584.2282, 586.2433, 597.2966, 598.2440, **600.2590,** 614.2745, 625.2904, 639.2698, 642.2931	387.1223, 433.1428, 451.1534, 461.1375, 479.1481, 483.1795, 493.1635, 511.1742, 525.1898, 540.2369, 558.2473, 568.2318, 582.2478, 585.2345
24	243.1014, 271.0964, ***285.1122,*** 303.1228, 317.1382, 329.1382	254.0936, 270.0885
25	107.0488, 137.0594, 143.0489, 145.0645, 151.0750, 175.075, 192.1015, 237.0909, ***269.1173***	107.0488, 137.0594, 145.0645, 175.0750, 237.0908
26	161.0833, 192.1018, 254.0937, 269.1173, 281.1174	[***192.1018***] 161.0832, 177.0881; **[*269.1173*]** 209.0951, 237.0900, 238.0979, 241.0849, 254.0927; [***281.1174***] 249.0901, 250.0979, 253.1214, 266.0928
27	285.1123, 295.0966, ***313.1073,*** 340.1542	223.0594, 253.0857, 281.0808, 285.0757
28	137.0594, 143.0488, 151.0750, 175.0750, 235.0751, 267.1015, ***299.1280,*** 301.1434, 345.1886	137.0593, 151.0751, 175.0749, 235.0750, 267.1013
29	151.0749, ***178.0858,*** 265.0857, 283.1328, 297.1120, 313.1306	118.0648, 146.0596, 163.0624
30	293.0807, **325.1069,** 339.1223	237.0910, 265.0858, 282.0886, 293.0809, 297.1121, 307.0966, 310.0834
31	265.0857, 279.1014, ***297.1122,*** 311.1273	237.09067, 265.08597, 282.0884
32	296.1283, 298.1075, ***313.1311***	280.09671, 298.1075
33	137.0593, 151.0750, 157.0645, 181.0644, 187.0748, 190.0858, 192.1014, 201.0907, 209.0595, 213.0907, 223.0751, 239.0701, 241.0857, 245.1170, 255.1014, 267.1015, 270.1487, 273.1118, 280.1330, 298.1439,315.1461	[***209.0595***] 181.0638; [***213.0907***]181.0638; [**223.0752**] 195.0794, 208.0505; [***241.0857***] 153.0326, 165.0252, 181.0666, 209.0592, 213.0907; [***267.1015***] 235.0739; **[270.1487]** 181.0638, 239.1054, 255.1242; [***298.1439***] 157.0644, 181.0647, 187.0749, 201.0907, 209.0595
34	151.0747, 192.1015, 207.0742, 211.0751, 233.0586, 237.0908, 239.0701, 243.1011, 253.0856, 265.0859, 268.1332, 271.0963, 282.1121, 285.1182, 296.1279, 297.1121, 313.1307	**[*239.0701*]**207.0431, 211.0743; [***265.0859***] 233.0594, 237.0906; **[*285.1182*]**197.0952, 221.0588, 225.0901, 242.0928, 253.0850, 257.1164, 270.0873
35	300.0630,***315.0865***	223.0388, 255.0651, 300.0628
36	263.0703, ***295.0971,*** 308.1281	245.0596, 263.0701, 277.0857
37	107.0488, 137.0595, 143.0489, 145.0645, 151.0750, 157.0645, 175.0749, 194.1173, 237.0908, 239.1064,***269.0908,***271.1328	107.0486, 137.0594, 145.0643, 175.0749, 209.0957, 237.0906, 251.1064
38	265.0859, ***297.1124***	233.0594, 237.0907, 265.0859, 282.0883
39	279.1072, 293.0117, 299.1272,***311.1276,***325.1429	248.0830, 279.1016, 296.1040

40	269.0808, 330.0735, ***345.0965***	269.0808, 285.0757, 295.0601, 299.0930
41	336.1229, ***353.1256***	336.1227
42	279.0889, 294.1124, **307.0841**	251.0384, 279.0889
43	280.0969, **309.0999,** 324.1227	281.1046, 294.0763
44	279.0889, 294.1124, **307.0841**	251.0384, 279.0889
45	280.1094, 296.1043, **311.1280**	280.1095, 296.1042
46	***329.1018***	255.0649, 314.078
47	294.1123,322.1076, ***323.1151***	279.0890, 295.1205, 308.0919
48	294.1123, 322.1076, ***323.1151***	279.0890, 295.1205, 308.0919
49	294.1123, 322.1076, ***323.1151***	279.0890, 295.1205, 308.0919
50	292.0969, 308.1283,***320.0920,***321.0996	290.0813, 282.0968, 318.0759
51	294.1123, 322.1076,***323.1151***	279.0890, 295.1205, 308.0919
52	308.1279, 336.1231, ***337.1306***	308.1279, 320.0916, 321.0992, 334.1072

Italic and bold in the MS^2 data represented the parent ion for the MS^3 data by Orbitrap MS.

Italic and bold in box in the MS^3 data represented the parent ion for the MS^3 data by Orbitrap, and followed was the MS^3 information

Table S2. MS^n information of the standards.

t_R (min)	Compounds [1]	*m/z*	$[M+H]^+$	ppm	MS^2 information	MS^3 information
16.66	Tetrahydroberberine	340.1539	$C_{20}H_{22}O_4N$	-0.405	150.0672, 165.0906,***192.1015***	148.0752, 177.0778, 192.1012
17.88	Tetrahydropalmatine	356.1854	$C_{21}H_{26}O_4N$	-0.245	150.0673, 165.0907,***192.1019***	148.0752,176.0702, 177.0779, 192.1014
19.62	Berberrubine	322.1072	$C_{19}H_{16}O_4N$	-0.204	***307.0832***	279.0888, 307.0834
21.13	Berberine	336.1227	$C_{20}H_{18}O_4N$	-0.295	292.0966, 320.0921,***321.0993***	290.0817,292.0966, 318.0755

[1]These standards were not detected in the *Sinomeniumacutum,* others who detected in the study were illustrated in the Table 1.
